# Prothrombotic and Inflammatory Markers in Elderly Patients with Non-Alcoholic Hepatic Liver Disease before and after Weight Loss: A Pilot Study

**DOI:** 10.3390/jcm10214906

**Published:** 2021-10-25

**Authors:** Antonio Gidaro, Roberto Manetti, Alessandro Palmerio Delitala, Emanuele Salvi, Luigi Bergamaschini, Gianpaolo Vidili, Roberto Castelli

**Affiliations:** 1 Department of Biomedical and Clinical Sciences Luigi Sacco, Luigi Sacco Hospital, University of Milan, Via G.B. Grassi N° 74, 20157 Milan, Italy; gidaro.antonio@asst-fbf-sacco.it (A.G.); emalele29@gmail.com (E.S.); luigi.bergamaschini@unimi.it (L.B.); 2 University of Sassari Department of Medical, Surgical and Experimental Science University Hospital of Sas-sari, Piazza Università N° 21, 07100 Sassari, Italy; rmanetti@uniss.it (R.M.); aledelitala@tiscali.it (A.P.D.); gianpaolovidili@uniss.it (G.V.)

**Keywords:** non-alcoholic fatty liver disease (NAFLD), insulin resistance, plasminogen activator inhibitor-1 (PAI-1), von Willebrand factor (VWF), factor VII (FVII), slimming, weight loss, TAFI, protein S

## Abstract

Background: Non-alcoholic fatty liver disease (NAFLD) is a pathological condition, ranging from fatty liver to chronic steatohepatitis (NASH), liver cirrhosis, and eventually to hepatocellular carcinoma. Recent findings suggest that patients with NAFLD have an increased risk of cardiovascular events and thromboembolism, which is independent of metabolic diseases that are frequently associated with NAFLD, such as diabetes, hyperlipidemia, and obesity. Methods: We evaluated 30 NAFLD patients, before and after weight loss. Plasma levels of C-reactive protein (CRP), fibrinogen, plasminogen activator inhibitor-1 (PAI-1), von Willebrand factor (VWF), homocysteine, coagulation protein S, Thrombin activable fibrinolysis inhibitor (TAFI), and factor VII (FVII) were assessed to evaluate whether they should be responsible of the prothrombotic state of NAFLD after weight loss. Results: At baseline, patients affected by NAFLD had a significantly higher levels of CRP, fibrinogen, PAI-1, VWF antigen, and FVII levels. After weight reduction, we observed a significant drop of inflammatory and prothrombotic markers, as well as glucometabolic, lipid profile. Conclusion: These findings provide evidence for a link between NAFLD/NASH and thromboembolism. The association seems to be linked with primitive thrombotic state and hypercoagulation due to increased levels of coagulation factors and reduced levels of PAI-1. This hypercoagulation state might explain increased levels of thrombosis and splanchnic thrombosis observed in NASH correlated cirrhosis.

## 1. Introduction

Non-alcoholic fatty liver disease (NAFLD) is a pathological condition, ranging from fatty liver (FL) to chronic steatohepatitis (NASH), liver cirrhosis, and eventually to hepatocellular carcinoma (HCC) [1].

Nonalcoholic fatty liver disease (NAFLD), on its whole spectrum of conditions ranging from steatosis to steatohepatitis (nonalcoholic steatohepatitis; NASH) and cirrhosis, is the most frequent liver disease in developed countries, now regarded as the liver manifestation of the metabolic syndrome [2]. Several studies indicate that NAFLD, especially in its necro-inflammatory form (NASH), is associated with a systemic proinflammatory/prothrombotic state, leading to atherothrombosis such as cardiovascular disease. The pathogenesis seems to be mediated through systemic release of proinflammatory and procoagulant factors from the steatosis liver (C-reactive protein, plasminogen activator inhibitor-1, interleukin-6, fibrinogen, and other proinflammatory cytokines) and is responsible for immunomodulated thrombosis [3,4,5,6,7]. Strong epidemiological, biochemical, and therapeutic evidences support the hypothesis that the primary pathophysiological derangement in most patients with NAFLD is insulin resistance. Insulin resistance leads to increased lipolysis, triglyceride synthesis, increased hepatic uptake of free fatty acids (FFA), and accumulation of hepatic triglycerides [8].

Fat-derived hormones, such as adiponectin, leptin, and resistin, are important co-regulators of hepatic insulin sensitivity. Insulin resistance may lead to activation of the coagulation cascade via increased levels of plasminogen activator inhibitor 1 (PAI-1) and factor VIII, whereas anticoagulant levels of protein Care decreased [9]. The topic, however, remains matter of debate and there are no consistent epidemiological or clinical data to support increased rates of venous thrombosis in NASH [10,11,12,13]. Aggressive lifestyle modifications focused on weight reduction and increased physical activities may reduce the inflammatory state of NAFLD, the insulin resistance, and the prothrombotic state which characterize NAFLD [14,15,16,17,18]. The vascular involvement of NAFLD might be considered its systemic burden, conditioning higher mortality in patients affected by the disease. High physical activity (PA) levels or exercise training (ET) should be a pivotal role of any treatment plan for obese individuals regardless of weight loss goals, and is associated with numerous CV benefits [14,15,16,17,18]. On the other hand, physical inactivity, obesity and disability correlate with institutionalization and loss of independence [19].

The aims of this study are to evaluate the parameters of the coagulation, fibrinolysis and inflammation in a group of NAFLD patients and to verify whether dietary modification or lifestyle change such as increased physical activity affects these parameters

## 2. Materials and Methods

### 2.1. Study Population

From 2 February to 5 May 2020, we prospectively enrolled patients with: (1) Age over 60 years, (2) certain diagnosis of NAFLD defined by: (i) Demonstration of hepatic steatosis by abdominal ultra-sonography or liver biopsy. Abdominal ultra-sonography was preferred to CT scanning due to its capability to detect mild to moderate steatosis. According to the general guidelines [19], we performed liver biopsy with the following indications: to confirm or exclude the diagnosis of NAFLD when ultra-sonography was not conclusive, to diagnose other liver diseases, and to determine amounts of damage to the liver when necessary for treatment and prognosis. The last includes necro inflammatory activity, which is potentially reversible, and collagen deposition with varying degrees of remodeling, which is potentially less reversible. (ii) Exclusion of significant alcohol consumption (<140 g/week. For men and <70 g/week for women). (iii) Exclusion of other causes of hepatic steatosis including drug-induced steatosis. (iv) Absence of coexisting chronic liver disease. 

The exclusion criteria were: (1) Ongoing therapy with at least one between: anti-inflammatory, anti-fibrinolytic, anti-coagulant, anti-platelet, anti-diabetic drugs, or anti-hypertensive agents. (2) History of smoking (active or previous), chronic obstructive pulmonary disease, renal failure, chronic inflammatory diseases, or neoplasms. (3) Non responders to diet and change of lifestyle, defined as a weight loss of less than 10% of the basal body weight after one year of follow-up. 

The study participants gave a written informed consent and local ethical committee approved the study (Ospedale Maggiore Policlinico di Milano; N 206 date 29 June 2013).

### 2.2. Sample Collection and Storage

Blood samples were collected after 12 h of fasting. Using sodium citrate 3.8% as an anti-coagulant, antecubital venous blood samples were drawn from patients affected by NAFLD, at baseline. Additional blood samples were collected when patients obtained 10% in decreased of basal body weight. Plasma was obtained by centrifuging the samples at 2000× *g* for 20 min at room temperature, frozen in small aliquots and stored at −80 °C until testing.

### 2.3. Measurements

Measurements included BMI, waist girth, and blood pressure. C reactive protein was measured by ELISA (Zymutest CRP, Hyphen BioMed, Andresy, France) with intra- and inter-assay coefficients of variation (CVs) of 7–11%. Fibrinogen was measured by a commercial coagulometric method (Diagnostica Stago, Asnières, France) with intra- and inter-assay CVs of 4% and 7%. Serum lipids were measured with the standardized methods of the Centers for Disease Control and Prevention, including total cholesterol, HDL cholesterol, and triglycerides. LDL cholesterol was estimated by the Friedewald equation [20]. When triglycerides were >400 mg/dL, LDL cholesterol was measured directly using an automated spectrophotometric assay. Glucose tolerance, insulin sensitivity, insulin secretion and insulin clearance were assessed by frequently sampled 75 g oGTT. Fasting serum glucose was determined using the hexokinase-glucose 6 phosphate dehydrogenase enzymatic assay, and insulin was determined using radioimmunoassay (Linc Research, St. Charles, MO, USA). Plasminogen activator inhibitor 1 (PAI-1) activity was measured using a commercial bio-immunoassay (Chromolize PAI-1, Bio pool, Umea, Sweden) with intra- and inter- assay CVs of 2.4% and 4.5%. Von Willebrand factor (VWF) antigen was measured in citrated plasma by an ‘‘in-house’’ sandwich ELISA using two monoclonal antibodies directed against different VWF epitopes (11B6.18 and 7G10.8). The intra- and inter-assay CVs were both 8%. Factor VII (FVII) activity was measured using a commercially available one-stage prothrombin time-based assay (Instrumentation Laboratory Company, Lexington, MA, USA) in accordance with the manufacturer’s instructions. The intra- and inter-assay CVs were 10%. Total homocysteine values were determined by high performance liquid chromatography (HPLC) with fluorometric detection and isocratic elution. Free protein S antigen was measured using the Asserachrom Free Protein S assay (Stago), a one-step ELISA assay that uses 2 monoclonal antibodies specific for distinct epitopes of the free form of protein S to directly measure free protein S in plasma. Thrombin-activatable fibrinolysis inhibitor (TAFI) was purified from fresh-frozen plasma by immunoaffinity chromatography followed by further purification on protein G-Sepharose and Q-Sepharose (Amersham Pharmacia Biotech, Uppsala, Sweden). TAFI antigen levels were determined by a sandwich-type enzyme linked immunosorbent assay using a monoclonal capturing antibody and a polyclonal detection antibody.

### 2.4. Physical Activity Quantification

The self-estimation of physical activity levels was assessed while using the German Physical Activity Questionnaire 50+ (GPAQ 50+) [21], a self-administered questionnaire assessing older adults’ PA level per week. 

### 2.5. Statistical Analysis

Kolmogorov–Smirnov test was performed to evaluate the normality of distribution of data. Quantitative data were expressed as mean, standard deviation, median and range. Student T-test and Mann–Whitney test (for non-parametric data) were used for comparison between groups. *p*-value less than 0.05 was considered statistically significant. Data are shown as median values and interquartile ranges (25th and 75th percentiles). The between-group differences were analyzed using Mann–Whitney non-parametric tests for independent samples, and the effects of weight loss were assessed using multivariate analysis of variance, Friedman’s test and Wilcoxon’s test for paired samples. Data were analyzed using the SPSS PC statistical package, version 17.00 (SPSS Inc., Chicago, IL, USA). Univariate analysis of coagulation, metabolic parameter was performed to assess relationship between obesity, NAFLD, physical activity and prothrombotic state. 

## 3. Results

During the observational period, sixty-five patients with certain diagnosis of NAFLD were evaluated, but only 30 met the inclusion criteria. In particular, 30 patients were excluded because ongoing therapy or history, five were non responders to diet and change of lifestyle, reducing the eligible number to 30 patients, (nine females and 21 males). 

Median age of the examined population was 72 years (IQR 70–73) (Table 1). Thirteen patients had NASH, two patients had liver cirrhosis (all those 15 patients performed liver biopsy before the study beginning), and 11 patients had hepatic steatosis. In our case series, liver biopsy was performed during observational time in four cases, showing NASH in two cases and liver cirrhosis in two cases. 

Among NAFLD patients, two were normal-weight, (BMI among 18.5 to < 25.0), five patients were over-weight (BMI among 25 to < 30), and 23 patients were obese having BMI > 30. 

BMI positively correlated with CRP (r = 0.57, *p* = < 0.0001), fibrinogen (r = 0.31, *p* = 0.0001), and PAI-1 levels (r = 0.75, *p* = 0.0001), vWF (r = 0.56, *p* = 0.001), F VII (r = 0.68, *p* = 0.001); basal glucose (r = 0.35, *p* = 0.0001), OGGT (r = 0.79, *p* = 0.0001), and TAFI (r = 0.49, *p* = 0.02). Waist circumference significantly correlated with basal glucose (r = 0.51, *p* = 0.0001), and OGTT (r = 0.45, *p* = 0.0001), systolic blood pressure (r = 0.49, *p* = 0.0001), and diastolic blood pressure (r = 0.51, *p* = 0.0001) as well as with coagulation parameters: PAI-1 (r = 0.52, *p* = 0.0001), vWF (r = 0.42, *p* = 0.0001), FVII (r = 0.53, *p* = 0.0001), TAFI (r = 0.29, *p* = 0.0001). 

Protein S was correlated to BMI (r = 0.46, *p* = < 0.0001), but not to waist circumference (r = 0.17, *p* = 0.19).

Both BMI and waist circumference were not correlated to homocysteine (respectively, r = 0.07, *p* = 0.58; r = 0.06, *p* = 0.67).

**Figure 1:**
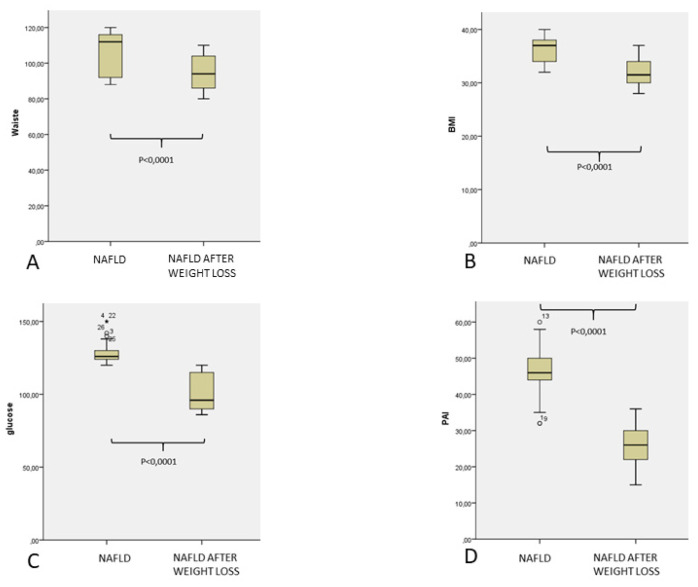
(**A**) Waist circumference. (**B**) body mass index. (**C**) glucose. (**D**) PAI 1 in 30 patients affected by NAFLD in basal control and after weight loss.

**Figure 2:**
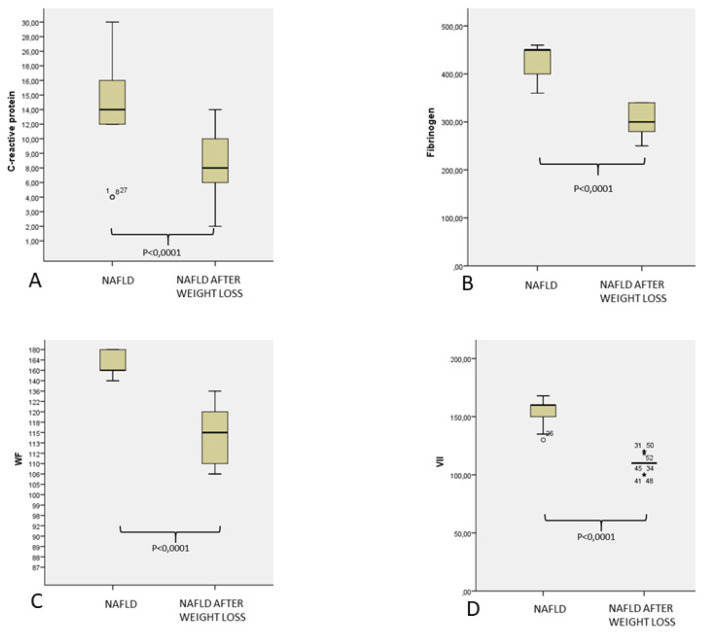
Plasma C-reactive protein levels (**A**), fibrinogen (**B**), von Willebrand factor (**C**), and factor VII levels (**D**) in 30 patients affected by NAFLD in basal control and after weight loss.

**Figure 3:**
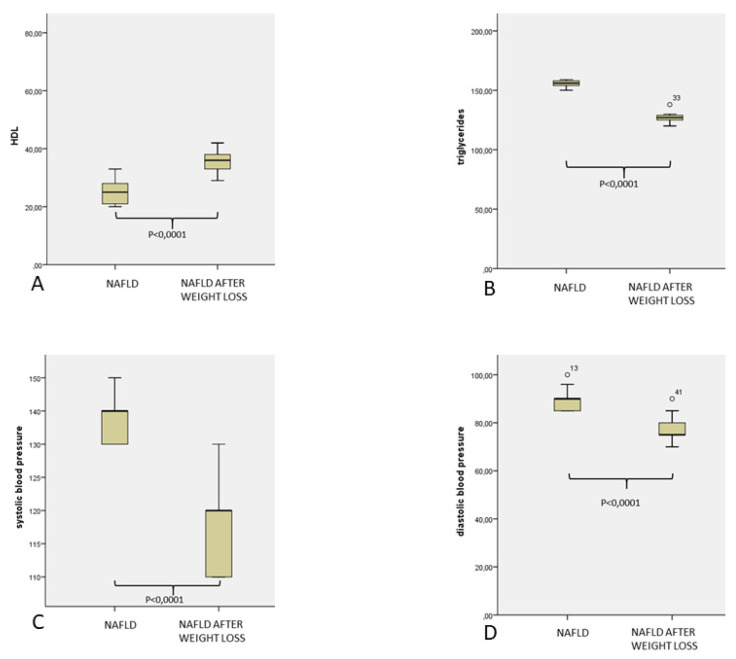
HDL cholesterol (**A**), triglycerides (**B**), systolic blood pressure (**C**), and diastolic blood pressure (**D**) of 30 patients affected by NAFLD in basal control and after weight loss.

After lifestyle modification was reached after one year of follow up, which was focused on a weight reduction to at least 10% of the basal and increased physical activity, we observed a significant reduction of BMI (*p* = 0.0001), waist circumference (*p* = 0.0001), as well as glucometabolic (*p* = 0.0001), lipid (*p* = 0.0001), and inflammatory (*p* = 0.0001), and prothrombotic markers (*p* = 0.0001), Figure 1, Figure 2 and Figure 3.

In this time frame, BMI had decreased from 36.4 Kg/m^2^ (34.2–38.6) to 32 Kg/m^2^ (29.4–34.6) (*p* = 0.0001), waist abdominal circumference from 106.3 cm (94.6–118.1) to 95.3 cm (85.7–104.9) (*p* = 0.0001). Similarly, inflammatory markers, as well as coagulation parameters, significantly decreased after weight loss: CRP from 14.7 mg/dL (8.3–21.1) to 8.1 mg/dL (4.7–11.5) (*p* = 0.0001), fibrinogen from 429.9 mg/dL (400.4–459.5 mg/dL) to 301.5 (272.8–330.9) mg/dL (*p* = 0.0001), PAI 1 from 46.9 UI/mL (40.3–53.5) to 26.7 U/l (20.4–33), von Willebrand factor 163.7% (149.8%–177.2) to 115.5% (109.1–121.9) (*p* = 0.0001), factor VII from 155.6% (146.6–164.5) to 109.9% (103.6–116.3) (*p* = 0.0001).

TAFI value increased after slimming, from 65.6% (52–80) to 79.7% (74–88) (*p* = 0.0001).

Statistical regression analysis showed that at 12 months, the changes in BMI positively correlated with those of CRP (r = 0.46, *p* = 0.025), fibrinogen (r = 0.44, *p* = 0.026), PAI-1 levels (r = 0.47, *p* = 0.019), VWF (r = 0.47, *p* = 0.045). Statistical analysis revealed significant gender and BMI interaction effects.

## 4. Discussion

The relationship between NAFLD and venous thrombosis, particularly portal vein thrombosis (PVT), is described in a limited number of patients. [22]. Nevertheless, splanchnic venous thromboembolism greatly contributes to the outcome and clinical course of liver cirrhosis.

The clinical observation by Dentali et al. [23], that metabolic syndrome was diagnosed in 50.5% of patients with idiopathic vein thrombosis, underlies the pathogenetic role of the metabolic syndrome in venous thromboembolism, but the real incidence of thrombotic events among NAFLD remains relatively unexplored.

It has long been known that diabetes, obesity, and hypertension increase the prothrombotic risk [11], but, to the best of our knowledge, this is the first study aimed at correlating coagulation assessment in the NAFLD and offering an overview of the interactions between coagulation factors and fibrinolysis with inflammatory system in these setting.

The present study indicates that patients affected by NAFLD have elevated levels of CRP, fibrinogen, PAI-1, von Willebrand factors, and F VII, which are known to be associated with an increased risk of thrombosis. This further supports the in vivo data for a hypercoagulable state existing in NAFLD [7]. On the other side, this study suggests that systemic inflammation predisposes patients to endothelial dysfunction and thrombosis. Recent data [9] examining plasma for levels of procoagulants and anticoagulants in patients across the spectrum of NAFLD, including those with cirrhosis, support this. Although the exact clinical implications of this finding remain unclear, we postulate that the imbalance may be due to increased factor VIII and reduced protein C levels, which may lead to the downstream hallmark effects of adverse cardiovascular events. The relationship between coagulation and liver fibrosis in NASH patients remains to be explained.

Weight loss (at least 10% of basal body weight) induced by physical exercise is linked with reduction in inflammatory and coagulation parameters thus reducing the thrombotic risk. Similar findings were observed by a previous paper in obese patients undergoing gastric banding after weight loss [24].

Data from the present studies show a highly significant and direct correlation between fibrinogen, PAI-I, von Willebrand factor, and Factor VII, with morphometric parameters such as BMI and waist circumference. On the other side, we observed a significant reduction of coagulation factors and a decreased level of PAI-1 after weight loss induced by diet and physical exercise. Conflicting results have also been observed regarding fibrinogen levels in obese patients after dietary treatment [25].

The reduction of PAI-1 and von Willebrand factors (both markers of vascular/endothelial damage) after weight loss strongly suggests an improvement of the endothelial dysfunction, as already reported in a similar paper on metabolic syndrome and inflammation [26].

The reversibility of the damage remains to be explained. We cannot exclude that increasing coagulation factors may be due to the fact that they are acute phase proteins, in response to a chronic inflammatory state linked with metabolic syndrome. Similarly, NAFLD is regarded as an inflammatory condition capable of activating coagulation and impairing fibrinolysis. As a consequence, we observed significant increased levels of PCR with a statistically significant correlation with BMI and waist circumference.

Nevertheless, the high prevalence of NAFLD among idiopathic vein thrombosis, as observed by Dentali et al., corroborates the pathogenetic link between metabolic syndrome and thrombogenesis [23].

Our patients with NAFLD have higher levels of PAI-1. The link between PAI-1 and the metabolic syndrome with obesity was established many years ago [27], and increased PAI-1 level can be now considered a true component of the syndrome. The production of PAI-1 by adipose tissue, in particular by tissue from omentum, has been demonstrated and could be an important contributor to the elevated plasma PAI-1 levels observed in insulin resistant patients supporting the notion that PAI-1 can be a link between NAFLD, insulin resistance and cardiovascular disease [28,29].

The increase of TAFI values after slimming in our cohort confirm that data of Lisman et al. [30], which reported a deficiency of this inhibitor in cirrhosis patients.

Other gastroenterological autoimmune conditions (such as Celiac disease [31] and inflammatory bowel disease [32]) are at an increased risk of venous thromboembolism; the genetic link between those disease is well established. If the hypercoagulability is confirmed in a future study, the genetic landscape of NAFLD patients has to be investigated to discover a possible link.

Gut microbiota dysregulation plays a key role in the pathogenesis of nonalcoholic fatty liver disease (NAFLD) [33] and in hypercoagulability [34] through its metabolites. Therefore, the restoration of the gut microbiota and supplementation with commensal bacterial metabolites, due to diet and change of lifestyle during our study, can be of therapeutic benefit against the disease.

Another consideration on our series regards the age. There are few reports on NAFLD in the elderly population [35], although immobilization and comorbidities, well known hypercoagulability risk factors, greatly impact on the course of these patients [19].

The main limitation of this study is the small number of patients. This is the result of the highly selective criteria designed to ensure the absence of comorbidities and of the classification of NAFLD in different subtypes according to histologic findings.

## 5. Conclusions

The originality of the paper lies in the observation that NAFLD is correlated with increased prothrombotic and inflammatory factors, directly correlated with BMI and waist circumference, which decrease after weight loss induced by physical exercise.

The second key message is that weight loss is linked to amelioration blood pressure, metabolic control, and reduction of the prothrombotic state also in NAFLD. However, larger studies are needed to confirm the observation and establish differences in subgroups of NAFLD (steatosis; steatohepatitis (nonalcoholic steatohepatitis; NASH) and cirrhosis.

## Figures and Tables

**Table 1:** Reports coagulation, fibrinolysis and inflammatory parameters, basal and after 10% of weight loss secondary to increase of PA. Data are reported as “median (IQR)”.

	Before Weight Loss	After Weight Loss	*p* Value
age (years)	72 (70–73)		
Waist circumference (cm)	112.0 (91.5–116.0)	94.0 (86.0–105.0)	<0.0001
BMI (Kg/m^2^)	37.0 (34.0–38.0)	31.5 (30.0–34.0)	<0.0001
Blood glucose (mg/dL)	126.00 (124.00–130.00)	96.00 (90.00–115.75)	<0.0001
OGTT (mg/dL)	180.00 (180.00–185.00)	160.00 (145.75–174.00)	<0.0001
CRP (mg/dL)	13.0 (12.0–16.5)	8.0 (6.0–10.0)	<0.0001
Fibrinogen (mg/dL)	450 (400–450)	300 (280–340)	<0.0001
PAI (UI/mL)	46.0 (44.0–50.0)	26.0 (21.5–31.5)	<0.0001
vWF (%)	160 (160–180)	115 (110–120)	<0.0001
F VII (%)	160 (150–160)	110 (110–110)	<0.0001
TAFI (%)	65.00 (60.00–70.00)	79.00 (78–82.25)	<0.0001
Homocysteine (µmol/L)	16 (14–18)	20 (17–22)	0.006
Protein S (%)	45.0 (42.0–46.5)	48.0 (44.0–48.0)	0.021
HDL (mg/dL)	25.00 (21.00–28.00)	36.00 (32.75–38.00)	<0.0001
Triglycerides (mg/dL)	156.00 (153.75–158.00)	127.00 (125.00–129.00)	<0.0001
PAS (mmHg)	140 (130–140)	120 (110–120)	<0.0001
PAD (mmHg)	90.00 (85.00–91.25)	75.00 (75.00–80.00)	<0.0001

## Data Availability

The study data will be made available upon request to the corresponding author.
